# Production of recombinant HPV11/16 E6/E7-MBP-His_6_ fusion proteins and their potential to induce cytokine secretion by immune cells in peripheral blood

**DOI:** 10.1186/s12985-023-02281-y

**Published:** 2024-01-05

**Authors:** Mei-nian Xu, Mei-zhen Zhong, Si-ning Feng, Yan-qin Xu, Xiao-ming Peng, Kang Zeng, Xiao-wen Huang

**Affiliations:** Department of Dermatology, Nanfang Hospital, Southern Medical University, Guangzhou, China

**Keywords:** Human papillomavirus, E6 protein, E7 protein, Recombinant protein, Maltose binding protein, Cytokine

## Abstract

Human papillomavirus (HPV) infection poses a significant threat to public health worldwide. Targeting the function of HPV E6 and E7 proteins and activating the host immune response against these proteins represent promising therapeutic strategies for combating HPV-related diseases. Consequently, the efficient production of soluble, high-purity E6 and E7 proteins is crucial for function and host immune response studies. In this context, we selected the pMCSG19 protein expression vector for *Escherichia coli* to produce soluble MBP-His_6_ tagged HPV11/16 E6/E7 proteins, achieving relatively high purity and yield. Notably, these proteins exhibited low toxicity to peripheral blood mononuclear cells (PBMCs) and did not compromise their viability. Additionally, the recombinant proteins were capable of inducing the secretion of multiple cytokines by immune cells in peripheral blood, indicating their potential to elicit immune responses. In conclusion, our study offers a novel approach for the production of HPV11/16 E6/E7 fusion proteins with relatively high purity and yield. The fusing HPV11/16 E6/E7 proteins to MBP-His_6_ tag may serve as a valuable method for large-scale protein production in future research endeavors.

## Introduction

Human papillomavirus (HPV)infection is one of the most prevalent sexually transmitted diseases worldwide. Low-risk HPV types, particularly HPV6 and HPV11, are associated with genital warts, recurrent respiratory papillomatosis, and oral papillomas. Persistent infection with high-risk HPV types, such as HPV16 and HPV18, contributes to cervical, oropharyngeal, and cutaneous cancers [1]. The consistent and stable expression of E6 and E7 oncoproteins in HPV-positive cells is crucial for persistent HPV infection and the development of malignant phenotype [2, 3]. These oncoproteins have been the focus of numerous studies aimed at understanding the molecular mechanisms of HPV-induced carcinogenesis and the development of therapeutic vaccines and diagnostic tools [4, 5]. In this respect, the efficient production of soluble, high-purity E6 and E7 proteins is essential for advancing our understanding of HPV-associated diseases and fostering the development of innovative medical interventions to combat these illnesses.

Although eukaryotic expression systems, such as insect or mammalian cells, allow for proper protein folding and post-translational modifications, these systems are expensive, time-consuming, and lower yield [6]. Bacterial systems, such as *Escherichia coli*, offer several advantages for recombinant protein expression, including rapid growth, high yield, simplicity, ease of use, cost-effectiveness, and high-throughput capabilities. However, the production of soluble and pure HPV E6 proteins in bacteria poses significant challenges due to the protein’s structure. The main focus has been on the HPV16 type when producing E6 proteins in bacteria [7–10]. This protein possesses numerous cysteine residues, encouraging the formation of disulfide bonds [9]. The extensive formation of these bonds restricts the exposure of hydrophilic residues to the solvent, thereby reducing protein solubility and causing protein misfolding. This, in turn, leads to protein aggregation and the creation of insoluble protein complexes. Consequently, aggregates form during overexpression in cells and persistently increase during purification [8]. Similarly, when E7 protein is overexpressed in bacteria, it mainly exists as insoluble inclusion bodies due to overloading the host protein folding machinery [11, 12]. However, unlike the cysteine-rich E6 protein that is susceptible to forming disulfide bond bridges, the E7 protein’s structure exhibits enhanced solubility [13]. Generally, the overexpression of heterologous proteins in a host necessitates strategies to improve their solubility and facilitate their correct folding.

Fusion tags, such as glutathione S-transferase (GST) or maltose-binding protein (MBP), can improve the solubility of fusion proteins. In this strategy, the MBP tag, when fused to the N-terminus of the target protein, can significantly enhance its solubility, preventing aggregation and facilitating proper folding [14, 15]. This is particularly beneficial for proteins that are prone to forming inclusion bodies or are difficult to express in a soluble form. Although MBP could increase the solubility of HPV16 E6 protein, some proteins still inevitably aggregate [16, 17]. Moreover, the hydrophobic nature of E6 proteins can lead to protein aggregation and loss during chromatography steps [15, 18].

One way around this obstacle is to fuse the desired protein to a dual-affinity tag designed in tandem with a protease cleavage site. The combination of the MBP and hexahistidine (His_6_) tags provides complementary benefits in recombinant proteins [19]. Fusing MBP to the N-terminus of target proteins increases their solubility and expression levels in the host system, facilitating their purification and analysis in their native and functional state. The His_6_ tag enables easy purification of the recombinant protein using immobilized metal affinity chromatography (IMAC). The strong and specific interaction between the His_6_ tag and metal ions (e.g., Ni^2+^ or Co^2+^) allows for a rapid and selective purification process, resulting in a high degree of purity.

In this study, we inserted the E6 and E7 segments of HPV11 and HPV16 into the plasmid pMCSG19, a bacterial vector with an MBP-TVMV-6xHis-TEV leader, to obtain E6/E7 proteins with an MBP-His_6_ tag. We then expressed and purified the recombinant HPV11/16 E6/E7 proteins with a fused MBP-His_6_ tag in a prokaryotic expression system. Finally, we assessed their impact on the cytotoxicity, cell viability, and cytokine secretion by immune cells in peripheral blood.

## Materials and methods

### Expression of HPV11 E6-MBP-His_6_, HPV11 E7-MBP-His_6_, HPV16 E6-MBP-His_6_, and HPV16 E7-MBP-His_6_ proteins in *Escherichia coli* cells

The E6 and E7 gene sequences of HPV11 (low-risk) and 16 (high-risk) (Table 1) were obtained from the NCBI Nucleotide database (https://www.ncbi.nlm.nih.gov/nucleotide). The plasmid pMCSG19, a bacterial vector with an MBP-TVMV-6xHis-TEV leader, was chosen as the vector to express the E6 and E7 proteins. Homologous sequences complementary to the plasmid vector insertion site were added to the 5’ ends of the forward primers and reverse primers of HPV11/16 E6/E7 genes (Table 2). The primers were designed and validated using the NCBI Primer-BLAST server (https://www.ncbi.nlm.nih.gov/tools/primer-blast/). Initially, the primers with homologous sequences were employed to amplify the HPV11/16 E6/E7 genes via PCR. Next, the target products were purified using a Gel DNA Recovery Kit (Bioteke Corporation, Beijing, China) subjected to an ‘In Fusion’ reaction with the pMCSG19 plasmid (Huayueyang Biotechnology Ltd., Beijing, China). In this process, the homologous sequences of the target gene and the plasmid vector combined to form a recombinant plasmid. Subsequently, the PCR reaction was treated with the DpnI restriction enzyme and introduced into DH5α competent *Escherichia coli* using the heat shock method, as previously described [20]. Finally, the constructed plasmids were extracted from *E. coli* and confirmed by sequencing (Sangon Biotech, Shanghai, China).

**Table 1:** Gene and protein sequences of HPV11/16 E6/E7

Gene	Gene sequence	Protein Sequence	Molecular weight
*HPV11 E6*	ATGGAAAGTAAAGATGCCTCCACGTCTGCAACATCTATAGACCAGTTGTGCAAGACGTTTAATCTTTCTTTGCACACTCTGCAAATTCAGTGCGTGTTTTGCAGGAATGCACTGACCACCGCAGAGATATATGCATATGCCTATAAGAACCTAAAGGTTGTGTGGCGAGACAACTTTCCCTTTGCAGCGTGTGCCTGTTGCTTAGAACTGCAAGGGAAAATTAACCAATATAGACACTTTAATTATGCTGCATATGCACCTACAGTAGAAGAAGAAACCAATGAAGATATTTTAAAAGTGTTAATTCGTTGTTACCTGTGTCACAAGCCGTTGTGTGAAATAGAAAAACTAAAGCACATATTGGGAAAGGCACGCTTCATAAAACTAAATAACCAGTGGAAGGGTCGTTGCTTACACTGCTGGACAACATGCATGGAAGACTTGTTACCCTAA	MESKDASTSATSIDQLCKTFNLSLHTLQIQCVFCRNALTTAEIYAYAYKNLKVVWRDNFPFAACACCLELQGKINQYRHFNYAAYAPTVEEETNEDILKVLIRCYLCHKPLCEIEKLKHILGKARFIKLNNQWKGRCLHCWTTCMEDLLP	17406.29
*HPV11 E7*	ATGCATGGAAGACTTGTTACCCTAAAGGATATAGTACTAGACCTGCAGCCTCCTGACCCTGTAGGGTTACATTGCTATGAGCAATTAGAAGACAGCTCAGAAGATGAGGTGGACAAGGTGGACAAACAAGACGCACAACCTTTAACACAACATTACCAAATACTGACCTGTTGCTGTGGATGTGACAGCAACGTCCGACTGGTTGTGGAGTGCACAGACGGAGACATCAGACAACTACAAGACCTTTTGCTGGGCACACTAAATATTGTGTGTCCCATCTGCGCACCAAAACCATAA	MHGRLVTLKDIVLDLQPPDPVGLHCYEQLEDSSEDEVDKVDKQDAQPLTQHYQILTCCCGCDSNVRLVVECTDGDIRQLQDLLLGTLNIVCPICAPKP	10889.46
*HPV16 E6*	ATGCACCAAAAGAGAACTGCAATGTTTCAGGACCCACAGGAGCGACCCAGAAAGTTACCACAGTTATGCACAGAGCTGCAAACAACTATACATGATATAATATTAGAATGTGTGTACTGCAAGCAACAGTTACTGCGACGTGAGGTATATGACTTTGCTTTTCGGGATTTATGCATAGTATATAGAGATGGGAATCCATATGCTGTATGTGATAAATGTTTAAAGTTTTATTCTAAAATTAGTGAGTATAGACATTATTGTTATAGTTTGTATGGAACAACATTAGAACAGCAATACAACAAACCGTTGTGTGATTTGTTAATTAGGTGTATTAACTGTCAAAAGCCACTGTGTCCTGAAGAAAAGCAAAGACATCTGGACAAAAAGCAAAGATTCCATAATATAAGGGGTCGGTGGACCGGTCGATGTATGTCTTGTTGCAGATCATCAAGAACACGTAGAGAAACCCAGCTGTAA	MHQKRTAMFQDPQERPRKLPQLCTELQTTIHDIILECVYCKQQLLRREVYDFAFRDLCIVYRDGNPYAVCDKCLKFYSKISEYRHYCYSLYGTTLEQQYNKPLCDLLIRCINCQKPLCPEEKQRHLDKKQRFHNIRGRWTGRCMSCCRSSRTRRETQL	19187.28
*HPV16 E7*	ATGCATGGAGATACACCTACATTGCATGAATATATGTTAGATTTGCAACCAGAGACAACTGATCTCTACTGTTATGAGCAATTAAATGACAGCTCAGAGGAGGAGGATGAAATAGATGGTCCAGCTGGACAAGCAGAACCGGACAGAGCCCATTACAATATTGTAACCTTTTGTTGCAAGTGTGACTCTACGCTTCGGTTGTGCGTACAAAGCACACACGTAGACATTCGTACTTTGGAAGACCTGTTAATGGGCACACTAGGAATTGTGTGCCCCATCTGTTCTCAGAAACCATAA	MHGDTPTLHEYMLDLQPETTDLYCYEQLNDSSEEEDEIDGPAGQAEPDRAHYNIVTFCCKCDSTLRLCVQSTHVDIRTLEDLLMGTLGIVCPICSQKP	11022.32

**Table 2:** Primers for inserting the E6 or E7 segments into plasmid pMCSG19 using ‘In-fusion’ PCR

Gene	Sequence of forward primer (5′→3′)	Sequence of reverse primer(5′→3′)
*HPV11 E6*	AGAACCTGTACTTCCAATCCATGGAAAGTAAAGATGCCT	CAGTGGTGGTGGTGGTGGTGGGGTAACAAGTCTTCCATG
*HPV11 E7*	AGAACCTGTACTTCCAATCCATGCATGGAAGACTTGTTA	CAGTGGTGGTGGTGGTGGTGTGGTTTTGGTGCGCAGATG
*HPV16 E6*	AGAACCTGTACTTCCAATCCATGCACCAAAAGAGAACTGC	CAGTGGTGGTGGTGGTGGTGCAGCTGGGTTTCTCTACGTG
*HPV16 E7*	AGAACCTGTACTTCCAATCCATGCATGGAGATACACCTAC	CAGTGGTGGTGGTGGTGGTGTGGTTTCTGAGAACAGATGG

The underlined sections represented homologous sequences complementary to the plasmid vector insertion site, which were added to the 5′ ends of the forward primers and reverse primers of the target genes.

Target plasmids were transformed into BL21(DE3) competent *E. coli* cells for protein expression, and the empty plasmid pMCSG19 was transformed as a control group. The cells were cultivated in an LB medium containing 100 µg/mL ampicillin in a shaking incubator at 37 °C. When the optical density of the medium reached 0.6 at 600 nm, isopropyl-β-D-thiogalactopyranoside (IPTG) was added at a final concentration of 0.1 mM to induce protein expression. After 5 h of induction, the cells were harvested by centrifugation and washed with cold PBS.

### Purification of the MBP-His_6_ tagged HPV11/16 E6/E7 proteins

For protein purification, the cells were re-suspended in PBS and disrupted by sonication. The supernatants were applied to an equilibrated Ni-nitrilotriacetic acid (NTA) resin column (Sangon Biotech, Shanghai, China), a nickel-charged affinity resin for affinity purification of His-tagged fusion proteins. Then, we performed a stepwise elution using different concentrations of imidazole (20 mM, 50 mM, and 300 mM) to optimize the elution conditions for both the MBP-His_6_ tag and the HPV11/16 E6/E7-MBP-His_6_ fusion proteins. The optimal imidazole concentration for eluting the MBP-His_6_ tag was 50 mM, while the HPV11/16 E6/E7-MBP-His_6_ fusion proteins were best eluted with 300 mM imidazole. These specific concentrations were chosen because they provided the best balance between protein purity and yield, as assessed by sodium dodecyl sulfate-polyacrylamide gel electrophoresis (SDS-PAGE). The potential endotoxin in the elution fraction was removed by flowing through polymyxin B-agarose (Sigma-Aldrich, St. Louis, Missouri, USA) and then detected by Chromogenic LAL Endotoxin Assay Kit (GenScript, Nanjing, China) following the manufacturer’s instructions. Next, the buffer of purified proteins was exchanged with sterile PBS and concentrated using Amicon Ultra centrifugal filters. Finally, the concentrations of the purified proteins were determined using a Pierce bicinchoninic acid (BCA) protein assay kit (Invitrogen, Carlsbad, CA, USA). The expression and purity of the proteins were validated by SDS-PAGE followed by Coomassie Brilliant Blue Staining as described in a previous study [21]. The recombinant proteins were stored in liquid nitrogen.

### Isolation and culture of peripheral blood mononuclear cells

Human peripheral blood mononuclear cells (PBMCs) were isolated from blood samples using the Isopaque-Ficoll method (TBD, Tianjin, China) according to the manufacturer’s instructions. Blood samples were collected from healthy volunteers, and informed consent was obtained from all subjects involved in the study. PBMCs were cultured at 1 × 10^6^ cells/mL density in RPMI-1640 medium (Invitrogen, Carlsbad, CA, USA) supplemented with 10% Fetal Bovine Serum (FBS; Invitrogen, Carlsbad, CA, USA) in a humidified incubator at 37 °C with 5% CO_2_.

### Stimulation of PBMCs with the recombinant HPV11/16 E6/E7-MBP-His_6_ proteins

PBMCs were isolated on the day of the experiment and plated on a 96-well plate at a density of 1 × 10^5^ cells/well. The recombinant proteins of HPV11 E6-MBP-His_6_, HPV11 E7-MBP-His_6_, HPV16 E6-MBP-His_6_, and HPV16 E7-MBP-His_6_ were diluted with sterilized PBS and added to the wells at the final concentrations of 1, 10, and 100 µg/mL, respectively. MBP-His_6_ protein treatments at corresponding concentrations were performed as controls. Then, the supernatants were collected at 24 h post-stimulation for further detection of secreted cytokines.

### Challenging mice with recombinant HPV11/16 E6/E7-MBP-His_6_ proteins

The animal experiment was conducted according to the Animal Care and Use Procedure (ACUP) guidelines. Specific pathogen-free (SPF) BALB/c mice (6–8 weeks old) were challenged by the tail vein injection with the recombinant proteins. The mice were housed in cages under SPF conditions with a natural light-dark cycle, a temperature of 21 °C, relative humidity between 40 and 60%, and food and water ad libitum in the Experimental Animal Center of Nanfang Hospital, Southern Medical University. Mice were administered a single intravenous injection of the recombinant HPV11 E6-MBP-His_6_, HPV11 E7-MBP-His_6_, HPV16 E6-MBP-His_6_, HPV16 E7-MBP-His_6_, and MBP-His_6_ proteins, with each mouse receiving 200 µg of protein. The experiment was conducted three times, with at least five mice per group in each repetition. Blood samples were collected from the orbital venous plexus of the mice 24 h post-injection, and serum was isolated from the blood for the detection of secreted cytokines.

### Evaluation of the impact of recombinant proteins on cytotoxicity of PBMCs

The cytotoxicity of recombinant HPV11/16 E6/E7-MBP-His_6_ proteins was determined using lactate dehydrogenase (LDH) assay kits (Beyotime, Shanghai, China). In detail, the cell supernatants were collected after 24 h of the stimulation with recombinant proteins and then subjected to the detection of LDH activity according to the manufacturer’s instructions. Cytotoxicity (%) = [(Protein-treated LDH activity – PBS-treated LDH activity) / (Maximum LDH release activity – PBS-treated LDH activity)] × 100.

### Evaluation of the impact of recombinant proteins on PBMC viability

The PBMC viability was measured by the CCK-8 kit (Fude Biological Technology, Hangzhou, China). Briefly, 10 µL CCK-8 solution was added to each well for a further 3 h at 37 °C. The optical density (OD) values of the reactant were measured at 450 nm wavelength using a spectrophotometer (ELX800, BioTek Instruments, Winooski, USA). Six replicated wells were carried out for each experiment. The cell viability was calculated by the following: cell viability (%) = [(OD experiment -OD blank) / (OD control – OD blank)] × 100. The experiments were repeated in triplicate with three replicates each.

### Evaluation of the impact of recombinant proteins on cytokine secretion of PBMCs and mouse serum

The concentration of 12 cytokines (IFN-γ, TNF-α, IL-2, IL-4, IL-5, IL-6, IL-9, IL-10, IL-13, IL-17 A, IL-17 F, and IL-22) in the culture supernatants of PBMCs and mouse serum was quantified by LEGENDplex Human Th cytokine panel (BioLegend, San Diego, CA, USA) on the flow cytometer (BD LSRFortessa, Franklin Lakes, USA) under close compliance with the manufacturer’s guidelines. The experiments were repeated in triplicate with three replicates each.

### Statistical analysis

Each experiment was repeated in triplicate. Data were analyzed using the SPSS software 15.0 (SPSS Inc., Chicago, IL, USA). The analytes for cytokine levels of each group were compared using a one-way ANOVA test with Bonferroni’s correction. The results were expressed as the means ± standard errors of the means (SEM), and the differences were considered significant when *p* < 0.05.

## Results

### Recombinant HPV11/16 E6/E7-MBP-His_6_ proteins were produced in a prokaryotic expression system

**Fig. 1:**
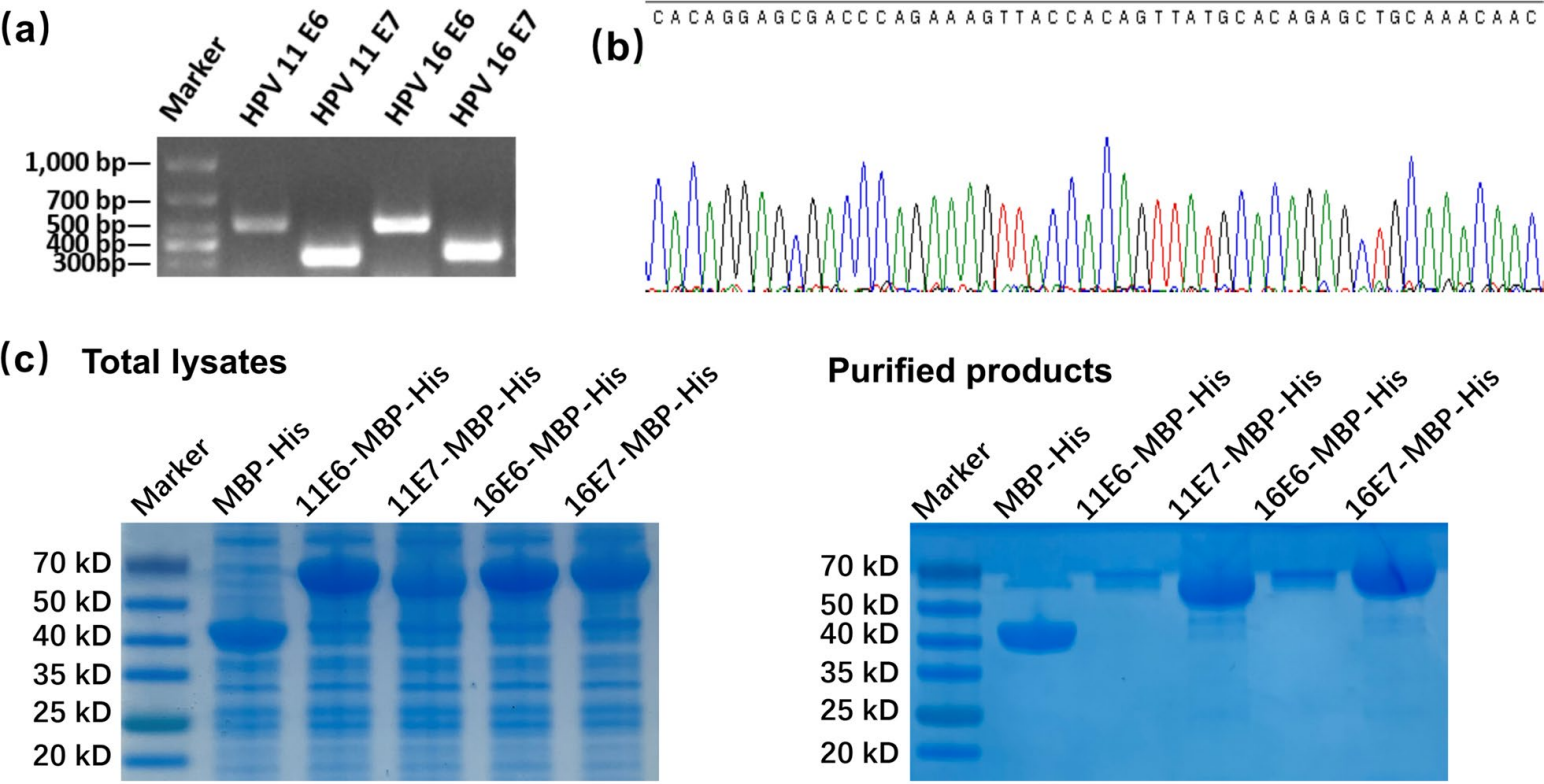
Recombinant and purification of HPV11/16 E6/E7-MBP-His_6_ proteins. **a** PCR products of HPV11 E6, HPV11 E7, HPV16 E6, and HPV16 E7 were visualized using gel electrophoresis. **b** Nucleotide sequence analysis for target sequences from the recombinant vectors. **c** The target protein bands were detected in the total lysates of *E. coli* cells (left) and purified products (right) using sodium dodecyl sulfate-polyacrylamide gel electrophoresis (SDS-PAGE), followed by Coomassie Brilliant Blue Staining

HPV11/16 E6/E7 was inserted into the C-terminus of the MBP-His_6_ tag in the *E. coli* plasmid pMCSG19 by In-fusion PCR. Primers with homologous sequences complementary to the plasmid vector insertion site were used to amplify the HPV11/16 E6/E7 genes via PCR. Electrophoresis result revealed that the PCR product sizes corresponded to the expected sizes of HPV11 E6 (493 bp), HPV11 E7 (337 bp), HPV16 E6 (517 bp), and HPV16 E7 (337 bp) (Fig. 1a). Nucleotide sequence analysis confirmed the accuracy of target sequences from the recombinant vectors (Fig. 1b). Target proteins were detected in the total lysates of *E. coli* cells by SDS-PAGE. The protein bands of MBP-His_6_, HPV11 E6-MBP-His_6_, HPV11 E7-MBP-His_6_, HPV16 E6-MBP-His_6_, and HPV16 E7-MBP-His_6_ proteins were located approximately at 43 kDa, 61 kDa, 54 kDa, 62 kDa, and 54 kDa regions, respectively (Fig. 1c, left). The thick protein bands suggested their abundant production and low cytotoxicity to the host cells. Then, the target proteins were purified. Endotoxin concentrations of the purified proteins were below 0.1 EU/mL. High purity of production was validated by SDS-PAGE analysis, presenting as a major band with the expected molecular weight in each lane (Fig. 1c, right). The thin bands of 11/16 E6-MBP-His_6_ proteins in SDS-PAGE implied relatively poor solubility, possibly due to inclusion bodies formation [17]. The procedure described above allowed a 4–6 mg yield in the case of 11/16 E6-MBP-His_6_ proteins and 24–30 mg in the case of 11/16 E7-MBP-His_6_ proteins from 1 L of cultured cells. Surprisingly, the yield of 11/16 E7-MBP-His_6_ proteins was close to that of MBP-His_6_. Still, the purified proteins were adequate for subsequent experiments. We attempted to remove the MBP-His_6_ tag, but the solubility of the target proteins significantly decreased, especially for the E6 proteins, resulting in a failure to obtain sufficient amounts of E6 and E7 proteins. This illustrates the importance of the MBP-His_6_ tag in maintaining the solubility of E6 and E7. In the subsequent experiments, we used the MBP-His_6_ tag as a control to clarify the cytotoxicity and immunogenicity of E6/E7 proteins in the fusion proteins.

### Recombinant HPV11/16 E6/E7 proteins had low toxicity to PBMCs and did not affect their viability

**Fig. 2:**
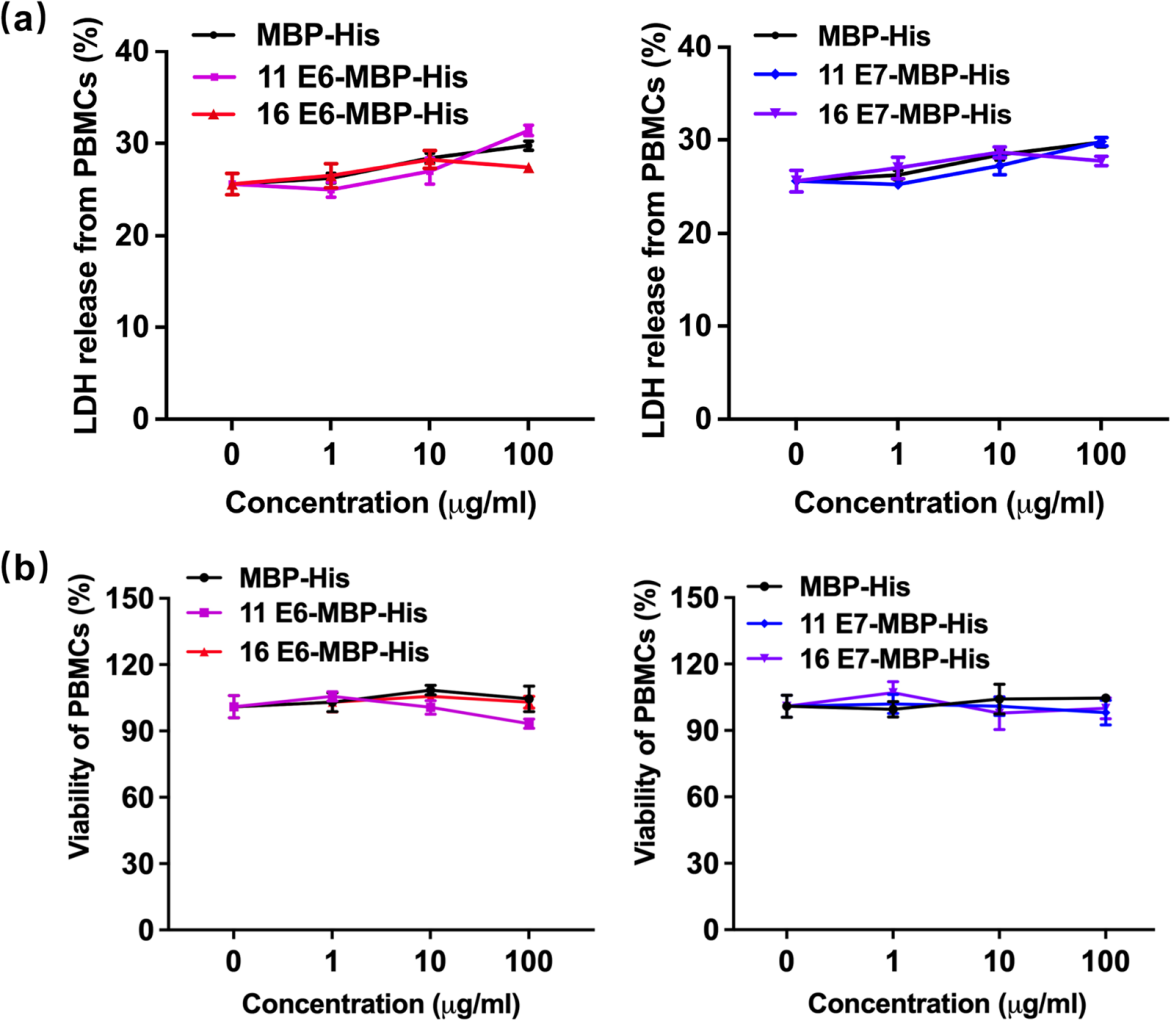
The impact of recombinant HPV11/16 E6/E7-MBP-His_6_ proteins on the cytotoxicity and cell viability of PBMCs. **a** The cytotoxicity of the recombinant proteins on PBMCs was evaluated using an LDH release assay. **b** The viability of PBMCs after the recombinant proteins treatment was detected by CCK-8 assay. Treatment with the MBP-His_6_ protein was used as the control. The experiments were done in triplicate, and data from representative ones were demonstrated

LDH release assay was performed to evaluate the cytotoxicity of HPV11/16 E6 and E7 proteins on PBMCs. As shown in Fig. 2a, 24 h after stimulating PBMCs with the recombinant proteins, LDH release slightly increased in a dose-dependent manner in all groups. In this context, all the treatments with recombinant proteins from 1 to 100 µg/mL resulted in approximate 26–30% increases in LDH activity. However, there was no significant difference in the LDH activity between HPV11/16 E6/E7-MBP-His_6_-treated groups and the MBP-His_6_-treated group. These results indicated that HPV11/16 E6/E7-MBP-His_6_ proteins had low toxicity to the peripheral immune cells.

Next, we detected the viability of PBMCs at 24 h after treatment with the recombinant proteins (1–100 µg/mL). Compared to the MBP-His_6_ treatment, HPV11/16 E6/E7-MBP-His_6_ treatments exhibited no significant impact on cell viability (Fig. 2b). Moreover, as the protein concentrations increased, there was also no significant change in cell viability. Thus, we suggested that the recombinant HPV11/16 E6 and E7 proteins had little effect on the viability of PBMCs.

### Secreted cytokine profiles in PBMCs after stimulation with recombinant HPV11/16 E6/E7 proteins

**Fig. 3:**
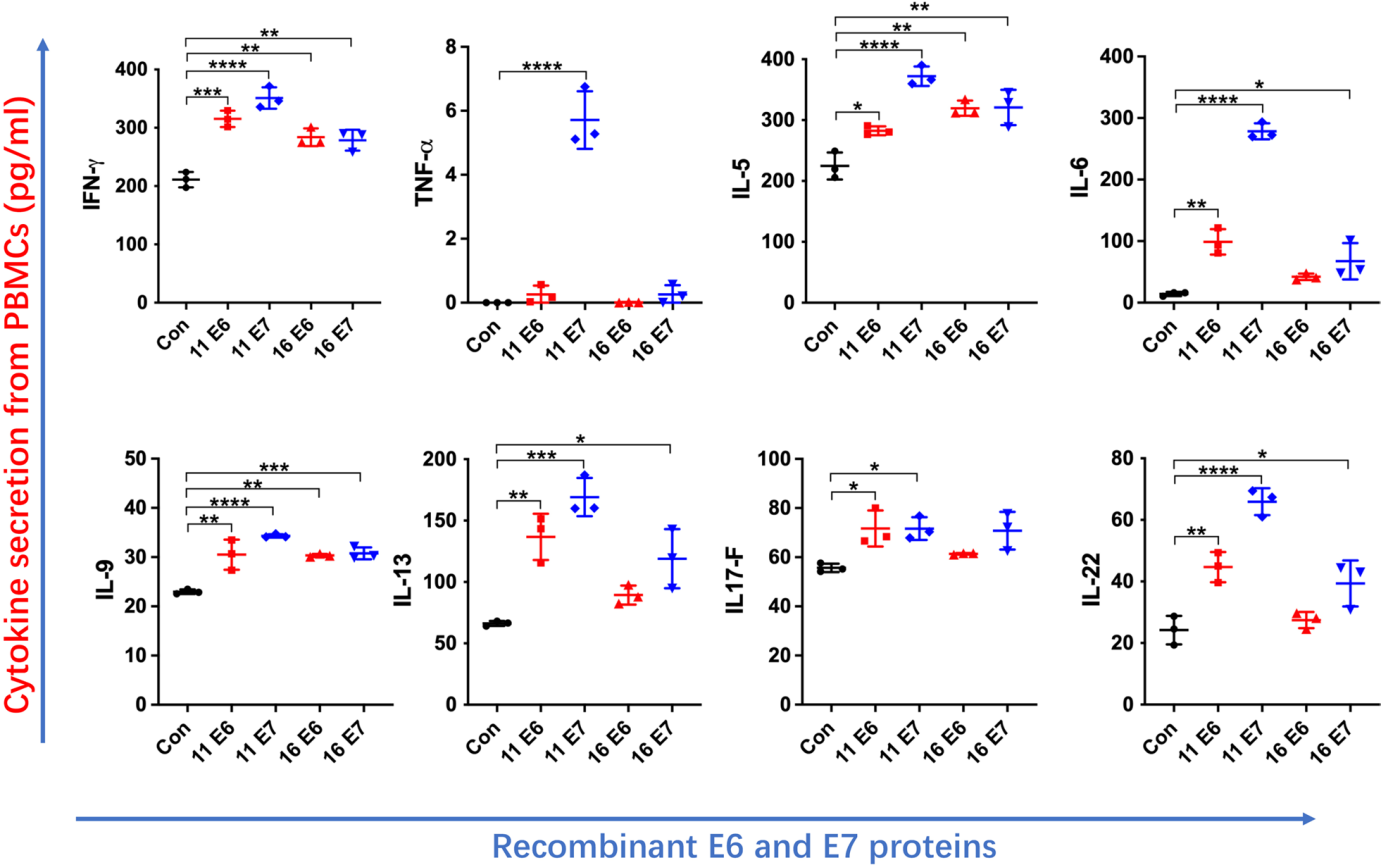
Cytokine expression of PBMCs after stimulation with recombinant HPV11/16 E6/E7-MBP-His_6_ proteins for 24 h. Cytokine profiles in the supernatants of PBMCs after stimulation with the recombinant HPV11/16 E6/E7-MBP-His_6_ proteins at a 10 µg/mL dose for 24 h. PBMCs with MBP-His_6_ stimulation were used as the control. The recombinant proteins of MBP-His_6_ and HPV11/16 E6/E7-MBP-His_6_ were abbreviated as Con, 11 E6, 11 E7, 16 E6, and 16 E7 in the figures. The data of IL-2, IL-4, IL-10, and IL-17 A were not shown due to the detection limitation. Values are means ± standard errors of the means (SEM). *, *p* < 0.05; **, *p* < 0.01; ***, *p* < 0.001; ****, *p* < 0.0001. One-way ANOVA test with Bonferroni’s correction.

Then we detected the secreted cytokine profiles in PBMCs at 24 h after stimulation with the recombinant proteins at the dose of 10 µg/mL [22]. MBP-His_6_ stimulation was performed as a control. As shown in Fig. 3, HPV11/16 E6/E7-MBP-His_6_ stimulation significantly upregulated the production of IFN-γ, IL-5, and IL-9 (*p* < 0.05) from PBMCs compared to MBP-His_6_ stimulation. PBMCs stimulated with HPV11 E6-MBP-His_6_, HPV11 E7-MBP-His_6_, and HPV16 E7-MBP-His_6_ promoted significantly increased production of IL-6, IL-13, and IL-22 (*p* < 0.05). Furthermore, the secretion of IL-17 F from PBMCs was significantly induced by stimulation with HPV11 E6-MBP-His_6_ and HPV11 E7-MBP-His_6_ (*p* < 0.05). The levels of IL-2, IL-4, IL-10, and IL-17 A were below the limit of detection (data not shown).

### Secreted cytokine profiles in the serum of mouse after being challenged with recombinant HPV11/16 E6/E7-MBP-His_6_ proteins

**Fig. 4:**
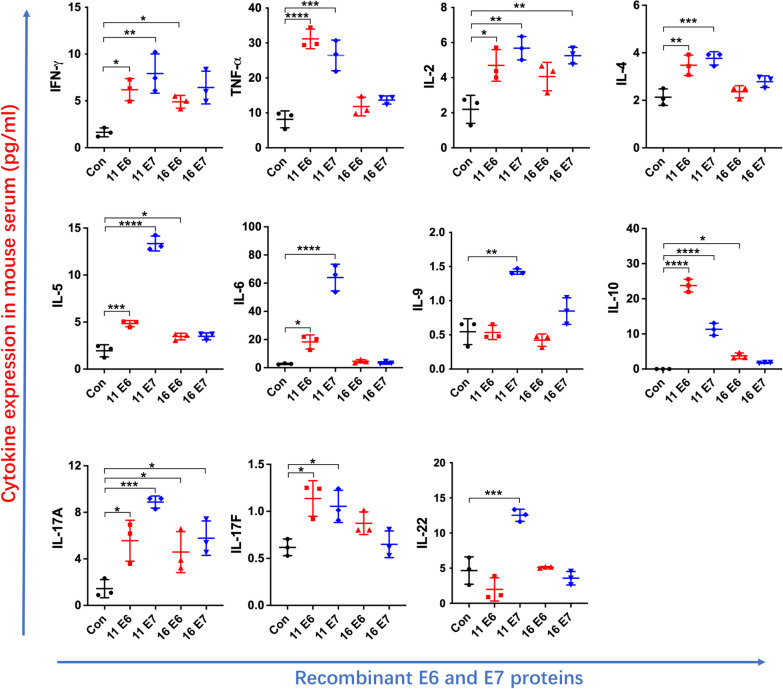
Cytokine expression in the serum of mice after immunization with recombinant HPV11/16 E6/E7-MBP-His_6_ proteins. Cytokine profiles in the serum of mice after being challenged with the recombinant HPV11/16 E6/E7-MBP-His_6_ proteins (200 µg per mouse) for 24 h. Mice challenged with MBP-His_6_ was used as the control. The experiment was repeated thrice with at least 5 mice per group. The recombinant MBP-His_6_ and HPV11/16 E6/E7-MBP-His_6_ proteins were abbreviated as Con, 11 E6, 11 E7, 16 E6, and 16 E7 in the figures. The data of IL-13 was not shown due to the detection limitation. Values are means ± standard errors of the means (SEM). *, *p* < 0.05; **, *p* < 0.01; ***, *p* < 0.001; ****, *p* < 0.0001. One-way ANOVA test with Bonferroni’s correction.

An in vivo experiment was performed to authenticate the impact of the recombinant HPV11/16 E6/E7 proteins on cytokine secretion. As shown in Fig. 4, compared with MBP-His_6_ challenge, the challenge with HPV11 E6-MBP-His_6_, HPV11 E7-MBP-His_6_, and HPV16 E6-MBP-His_6_ significantly upregulated the expression levels of IFN-γ, IL-5, IL-10, and IL-17 A in mouse serum (*p* < 0.05). Moreover, there were significantly higher expression levels of TNF-α, IL-2, IL-4, IL-6, and IL-17F upon the challenge with HPV11 E6-MBP-His_6_ and HPV11 E7-MBP-His_6_ than MBP-His_6_ challenge (*p* < 0.05). HPV11 E7-MBP-His_6_ protein challenge promoted significant expression of IL-9 and IL-22 from PBMCs compared to MBP-His_6_ challenge (*p* < 0.05). The level of IL-13 was below the limit of detection (data not shown).

## Discussion

In this study, we successfully fused HPV11 E6, HPV11 E7, HPV16 E6, and HPV16 E7 proteins to the MBP-His_6_ tag and obtained the target proteins with relatively high purity and yield. These proteins exhibited low toxicity to PBMCs and did not compromise their viability. Furthermore, they were capable of inducing the secretion of multiple cytokines by immune cells in peripheral blood, suggesting their bioactive potential in eliciting immune responses.

Recombinant protein expression technology is an essential tool for studying protein function, structure, vaccine synthesis, and screening targeted drugs. In the process of generating recombinant proteins, the N-terminal or C-terminal of the target protein is often fused and expressed with other specific proteins, peptides, or oligopeptide tags. This practice can not only retain the structure of natural proteins, but also increase solubility, prevent degradation, promote secretion, and facilitate purification [23]. It has been reported that MBP significantly improves solubility when fused to the N-termini of various target proteins, but this effect is considerably less pronounced when attached to their C-termini [14, 24, 25]. Besides, the His_6_ tag enables easy and efficient purification of the target protein using IMAC. In our study, the MBP-His_6_ was fused to the N-termini of E6 and E7 proteins. The dual-affinity tag system MBP-His_6_ enabled us to obtain soluble and pure proteins, which is essential for downstream functional and structural analyses. Specifically, the yield of MBP-His_6_-tagged E6 protein ranges from 4 to 6 mg per liter of cultured cells, whereas the yield of MBP-His_6_-tagged E7 protein reaches a higher level of 24 to 30 mg per liter of cultured cells. The yields of HPV11/16 E6-MBP-His_6_ proteins obtained by this method could fully meet general experimental needs.

After protein purification using IMAC, the concentration of soluble E7-MBP-His_6_ proteins remained abundant, while the concentration of soluble E6-MBP-His_6_ proteins was significantly reduced. This phenomenon may be attributed to the poor solubility and aggregation properties of E6 proteins in a recombinant way. When the E6 protein was efficiently expressed in prokaryotic cells, it tends to misfold and form inclusion bodies [26]. Some researchers have used a prokaryotic expression vector to fuse MBP with HPV16 E6 protein to improve solubility. Although they found that the solubility of E6 protein was enhanced, some proteins still inevitably aggregated [15, 27]. Inclusion body formation depends on the rate of protein folding and aggregation. Lowering the growth temperature of recombinant bacteria is the most common method to reduce inclusion body formation, but it is time-consuming [28]. We attempted to grow *E. coli* at room temperature for HPV11/16 E6-MBP-His_6_ expression but failed to significantly increase their solubility. Further research is needs to explore approaches to improve the solubility of E6 proteins. Additionally, we tried to remove the MBP-His_6_ tag and observed a dramatic decrease in the solubility of the target protein, particularly the E6 proteins. As a result, we were unable to obtain sufficient amount of proteins for our experiments. The highly soluble MBP plays a crucial role in preventing the precipitation of particles produced by E6. However, once the E6 protein is partially dissociated from MBP through protease hydrolysis, it precipitates instantly [8].

Considering that E6 and E7 proteins are critical targets in immunotherapy for HPV-related diseases, we measured the biological activity of E6/E7 proteins in the MBP-His_6_-fused HPV11/16 E6/E7 proteins, including their cytotoxicity and impacts on the cell viability and cytokine secretion by immune cells in peripheral blood. Consistent with the computational analysis of a previous study, E6 and E7 proteins exhibited low toxicity to the host immune system [29]. Interestingly, we observed a significantly increase in the secretion of multiple cytokines from PBMCs and mice serum after challenging with recombinant HPV11/16 E6/E7-MBP-His_6_ proteins. Notably, these cytokines are involved in both innate immune response and adaptive immune response. In this study, cytokine profiles in the in vivo and in vitro experiments were evaluated after 24 h, indicating an early immune response but not dynamic changes. However, the overall results support the potential biological activity of recombinant HPV11/16 E6/E7-MBP-His_6_ proteins.

Recent research has indicated that the MBP-His_6_ or MBP tag can enhance the immunogenicity of target protein [30–32]. This finding holds significant implications for the development of therapeutic vaccines using the MBP-His_6_ tag. The enhancement of immunogenicity by the MBP-His_6_ tag is primarily attributed to the properties of the MBP component. One contributing factor is the relatively large molecular weight of MBP, approximately 42 kDa. When fused with the target protein, MBP increases the molecular weight of the entire fusion protein, thereby improving its recognition by the immune system. Additionally, MBP, being an exogenous protein derived from *E. coli*, may be perceived as a non-self protein in mammals, which could trigger an immune response, behaving like an adjuvant. Furthermore, the stable structure of MBP aids in the correct folding of the target protein. Correctly folded proteins are more likely to be recognized as antigens by the immune system, thereby eliciting an immune response [33]. Lastly, MBP has the ability to enhance the solubility of the target protein, increasing its bioavailability within the body. Proteins with greater solubility are more readily recognized and processed by the immune system. Therefore, studying the impact of the MBP-His_6_ tag on the immunogenicity of the target protein may provide a promising direction for the development of HPV therapeutic vaccines. However, our study was limited by the unavailability of sufficient HPV11/16 E6/E7 proteins as a control for MBP-His_6_-tagged HPV11/16 E6/E7 proteins.

## Conclusions

Taken together, our research presents a new method for the production of MBP-His_6_-tagged HPV11/16 E6/E7 proteins with relatively high purity and yields, which possess the potential to induce cytokine secretion. The fusion of MBP-His_6_ tag to the HPV11/16 E6/E7 proteins may be used for large-scale protein production in the future. Future studies should focus on the effect of MBP-His_6_ tag on the immunogenicity of E6 and E7 proteins.

## Data Availability

The data that support the findings of this study are available from the corresponding author upon reasonable request.

## Abbreviations

HPV: Human papillomavirus
MBP: Maltose binding protein
PBMCs: Peripheral blood mononuclear cells
NTA: Ni-nitrilotriacetic acid
SPF: Specific pathogen-free
LDH: lactate dehydrogenase
SEM: standard errors of the means
